# S130. INCIDENCE OF FIRST EPISODE OF PSYCHOSIS IN AN AUSTRALIAN COHORT AND ASSOCIATIONS WITH NEIGHBOURHOOD CHARACTERISTICS

**DOI:** 10.1093/schbul/sby018.917

**Published:** 2018-04-01

**Authors:** Scott Eaton, Linglee Downey, Kristen Thien, Meghan Bowtell, Melissa Bardell-Williams, Aswin Ratheesh, Patrick McGorry, Brian O’Donoghue

**Affiliations:** Orygen, the National Centre of Excellence in Youth Mental Health

## Abstract

**Background:**

The incidence of psychotic disorders varies between geographical areas and is associated with neighbourhood characteristics. However, the research to date has been mainly confined to Northern European and North American populations. This study will determine whether the incidence of first episode psychosis (FEP) is associated with neighbourhood characteristics, specifically social deprivation, unemployment, social fragmentation and social capital.

**Methods:**

This study was conducted at the Early Psychosis Prevention and Intervention Centre (EPPIC) which provides specialist treatment to all young people aged 15–24 diagnosed with a FEP residing in a defined geographical catchment area within western and northwestern Melbourne. Census data was used to code postcodes for neighbourhood characteristics and determine the at-risk population of people aged 15–24 living within the catchment area. Incidence rate ratios were calculated.

**Results:**

527 young people treated for a FEP over a three-year period met inclusion criteria. This represents an annual incidence rate of 105.34 per 100,000 persons aged 15–24 per year. There was an increased incidence of FEP in neighbourhoods of greatest social deprivation (IRR=1.60, p=0.003), highest unemployment (IRR=1.67, p=0.001), least social capital (IRR=1.32, p=0.06) and above average social fragmentation (IRR=1.57, p=0.005). All these associations were stronger for non-affective psychoses and absent for affective psychoses. There was variation between sexes, with association only present for social fragmentation in women and social deprivation in men.

**Discussion:**

This study demonstrates that the incidence of psychotic disorders varies according to neighbourhood characteristics, with higher rates in neighbourhoods with higher inequality. Services in each area should be resourced appropriately to ensure that the expected incidence can be effectively managed.

